# Differential Effects of Parkinson's Disease and Dopamine Replacement on Memory Encoding and Retrieval

**DOI:** 10.1371/journal.pone.0074044

**Published:** 2013-09-26

**Authors:** Alex A. MacDonald, Ken N. Seergobin, Adrian M. Owen, Ruzbeh Tamjeedi, Oury Monchi, Hooman Ganjavi, Penny A. MacDonald

**Affiliations:** 1 The Brain and Mind Institute, University of Western Ontario, London, Ontario, Canada; 2 Department of Psychology, University of Western Ontario, London, Ontario, Canada; 3 Faculty of Law, University of Ottawa, Ottawa, Ontario, Canada; 4 Functional Neuroimaging Unit, Centre de Recherche, Institut Universitaire de Gériatrie de Montréal, Montreal, Quebec, Canada; 5 Department of Radiology, University of Montreal, Montreal, Quebec, Canada; 6 Department of Psychiatry, University of Western Ontario, London, Ontario, Canada; 7 Clinical Neurological Sciences, University of Western Ontario, London, Ontario, Canada; University of Chicago, United States of America

## Abstract

Increasingly memory deficits are recognized in Parkinson's disease (PD). In PD, the dopamine-producing cells of the substantia nigra (SN) are significantly degenerated whereas those in the ventral tegmental area (VTA) are relatively spared. Dopamine-replacement medication improves cognitive processes that implicate the SN-innervated dorsal striatum but is thought to impair those that depend upon the VTA-supplied ventral striatum, limbic and prefrontal cortices. Our aim was to examine memory encoding and retrieval in PD and how they are affected by dopamine replacement. Twenty-nine PD patients performed the Rey Auditory Verbal Learning Test (RAVLT) and a non-verbal analogue, the Aggie Figures Learning Test (AFLT), both on and off dopaminergic medications. Twenty-seven, age-matched controls also performed these memory tests twice and their data were analyzed to correspond to the ON-OFF order of the PD patients to whom they were matched. We contrasted measures that emphasized with those that accentuated retrieval and investigated the effect of PD and dopamine-replacement on these processes separately. For PD patients relative to controls, encoding performance was normal in the off state and was impaired on dopaminergic medication. Retrieval was impaired off medication and improved by dopamine repletion. This pattern of findings suggests that VTA-innervated brain regions such as ventral striatum, limbic and prefrontal cortices are implicated in encoding, whereas the SN-supplied dorsal striatum mediates retrieval. Understanding this pattern of spared functions and deficits in PD, and the effect of dopamine replacement on these distinct memory processes, should prompt closer scrutiny of patients' cognitive complaints to inform titration of dopamine replacement dosages along with motor symptoms.

## Introduction

Parkinson's disease (PD) is a neurodegenerative illness characterized by degeneration of the dopamine-producing cells of the substantia nigra (SN) [1]. The SN innervates the dorsal striatum, defined as the bulk of the caudate nuclei and putamen. The resulting dopamine depletion of the dorsal striatum in PD produces tremor, bradykinesia, and rigidity. Dopaminergic medications such as L-3,4-dihydroxyphenylalanine (L-Dopa) or dopamine receptor agonists improve these motor symptoms that typify PD [2].

Cognitive impairments are increasingly described in PD [3]. The nature and etiology of these deficits, as well as the effect of dopaminergic medications on these processes, however, are less clear [4]–[6]. Despite initial assumptions and occasional contradictory findings [7], [8], memory impairments are recognized in PD [9]–[12]. Successful remembering depends upon effectively a) acquiring or encoding new information and b) retrieving or accessing that information at a later time. These processes are dissociable by experimental manipulations [13]–[15]. During encoding, increased activity in medial temporal structures (e.g., hippocampus, perirhinal cortex) and decreased activity in regions of the default mode network (e.g, inferior parietal cortex, precuneus) have been shown [16]–[20]. In contrast, retrieval has been associated with increased activity in posterior parietal, anterior prefrontal, and posteromedial cortices [16], [21]–[23]. Inconsistencies in the literature with respect to memory performance in PD patients could owe to the fact that most studies examine only the combined effects of encoding and retrieval processes, in unknown proportions. Only one previous study aimed to distinguish these separable processes in PD [11]. Contradictory findings in PD possibly also result from comparing studies in which PD patients are tested off relative to on dopaminergic medication. Few studies have investigated the effect of dopamine replacement on memory in PD [10], [24] and none have systematically examined the effect of medication on encoding and retrieval processes separately. Hence, whether encoding and subsequent retrieval are differentially affected by PD and dopamine replacement remains unclear. Finally, the possibility that memory for verbal versus non-verbal materials differs in PD has not to this point been explored. Consequently, a comprehensive study, where the same group of PD patients is investigated, on and off dopaminergic therapy, with both verbal and non-verbal materials, using tests in which encoding and retrieval can be relatively controlled and distinguished is needed.

### Investigating the Effect of PD and Dopamine Replacement on Encoding Rate and Retrieval

The aim of the current study was to clarify memory performance in PD patients. Specifically, our aim was to investigate encoding and retrieval in PD patients, on and off dopamine replacement, relative to age-matched controls, using both verbal and non-verbal materials.

## Methods

### Participants

Twenty-nine PD patients with an average Hoen and Yahr staging of 1.96 (SEM 0.11) participated in the study. All patients were evaluated in a general neurology clinic, were diagnosed by a licensed neurologist, and met a) the core assessment program for surgical interventional therapy criteria for the diagnosis of idiopathic PD [25] and b) the UK Brain Bank criteria for the diagnosis of Parkinson's disease [26]. Twenty-seven age- and education-matched healthy control participants were also included in the current experiment. Patients and controls known for dementia or mild cognitive impairment, abusing alcohol, prescription or street drugs, or taking medications such as Donepezil, Rivastigmine, Galantamine, or Memantine were excluded from participation. Further, if patients described a change in function related to cognitive symptoms, performed below 100 on the Adult National Reading Test (ANART), or could not successfully draw a clock or copy a cube, they were excluded from the study. This study was approved by the Ethics Review Board of the Sudbury Regional Hospital and all participants provided informed written consent that was approved by the review board prior to testing according to the Declaration of Helsinki [27].

Severity and presence of disease were assessed for all patients both on and off dopaminergic medication using the motor sub-scale of the Unified Parkinson's Disease Rating Scale (UPDRS) by the senior author, a movement disorders neurologist. A screening neurological examination was performed on control participants and none manifested signs of PD. All patients and no controls were treated with L-Dopa. Eleven PD patients were also treated with pramipexole, a dopamine agonist medication. This medication constituted adjunctive therapy only, on average accounting for 25% of the daily L-Dopa medication equivalent. Mean group demographic information, screening affective and cognitive measures, UPDRS scores on and off medication, as well as daily doses of dopamine-replacement therapy in L-Dopa equivalents are presented in Table 1. Calculation of daily L-Dopa equivalent dose for each patient was based on theoretical equivalence to L-Dopa [28] as follows: L-Dopa dose + L-Dopa dose × 1/3 if on entacapone + bromocriptine (mg) × 10 + cabergoline or pramipexole (mg) ×67 + ropinirole (mg) × 20 + pergolide (mg) ×100 + apomorphine (mg) × 8.

**Table 1:** Demographics and clinical information, as well as screening cognitive and affective measures for PD patients and controls.

	PD	Control
N	29	27
Age	63.79 (1.61)	63.78 (1.43)
Education	13.72 (0.69)	12.67 (0.59)
Years Disease	5.02 (0.99)	–
LED (mg)	520 (67.32)	–
DA (n)	11	–
UPDRS ON	17.19 (1.43)	–
UPDRS OFF	21.76 (1.73)	–
BDI-II ON	7.87 (0.99)	4.19 (0.76)
BDI-II OFF	9.80 (1.33)	4.09 (0.71)
Apathy ON	11.76 (1.31)	8.89 (1.03)
Apathy OFF	11.70 (1.26)	8.67 (1.10)
ANART IQ	120.32 (1.47)	121.40 (1.32)
F-words	13.52 (1.09)	14.22 (1.12)
A-words	10.86 (0.63)	11.20 (0.54)
S-words	13.98 (0.74)	15.30 (0.63)
Animals	18.40 (0.70)	20.94 (0.78)
Clock	3 (0)	3 (0)
Cube	1 (0)	1 (0)
WCST Categories	3.76 (0.30)	4.39 (0.27)
WCST Perseverative Errors	17.81 (1.76)	16.78 (1.47)
WCST Non-perseverative Errors	21.03 (2.52)	15.63 (1.93)

Screening affective and cognitive measures are presented as group means (SEM). Control participants did NOT receive dopaminergic therapy during any session of the experiment. Their data are presented here to correspond to the ON-OFF order of the PD patient to whom they were matched.

**Education**  =  years of education; **Years Disease  = ** years since diagnosis of PD; **LED  = ** daily L-DOPA equivalent dose in mg; **DA  = ** number of patients taking dopamine agonists; **UPDRS ON  = ** Unified Parkinson's Disease Rating Scale motor score on medication; **UPDRS OFF  = ** Unified Parkinson's Disease Rating Scale motor score off medication; **BDI-II ON**  =  Beck Depression Inventory II score measured for PD patients while they were treated with their usual dopamine-replacement therapy and for control participants during the session that corresponded to the ON session of the PD patient to whom they were matched; **BDI-II OFF**  =  Beck Depression Inventory II score measured for PD patients while they abstained from their usual dopamine-replacement therapy and for control participants during the session that corresponded to the OFF session of the PD patient to whom they were matched; **Apathy ON**  =  Apathy Evaluation Scale score measured during the ON session; **Apathy OFF**  =  Apathy Evaluation Scale score measured during the OFF session; **ANART IQ  = ** National Adult Reading Test [95] IQ estimation tested in the ON session; **F-words**  =  average number of words beginning with the letter F generated in one minute in ON and OFF sessions; **A-words**  =  average number of words beginning with the letter A generated in one minute in ON and OFF sessions; **S-words**  =  average number of words beginning with the letter S generated in one minute in ON and OFF sessions; **Animals**  =  average number of animal names generated in one minute in ON and OFF sessions; **Clock  = ** score on clock drawing component of Montreal Cognitive Assessment (MOCA) tested in the ON session; **Cube  = ** score on cube copying component of MOCA tested in the ON session; **WCST Categories**  =  average number of correct categorizations on the Wisconsin Card Sorting Test (WCST) in ON and OFF sessions; **WCST Perseverative Errors**  =  average number of perseverative errors in the WCST in ON and OFF sessions; **WCST Non-perseverative Errors**  =  average number of non-perseverative errors in the WCST in ON and OFF sessions.

There were no statistically significant demographic differences between PD patients and controls and all participants performed normally on screening cognitive measures. Patients scored significantly higher on the Beck Depression Inventory–II (BDI-II) than controls but no participants were severely or even moderately depressed. We used a cut-off of 28/63 on the BDI-II as an a priori exclusion criterion but no patients approached this cut-off. Further, there were no differences in terms of the depressive symptoms endorsed by PD patients or controls between the on and off sessions.

### Apparatus

The experiment was conducted on a 12.1 inch widescreen laptop (Lenovo ThinkPad ×201) running at a resolution of 1280×800 on the Windows 7 operating system. The screen was angled for optimal viewing at a distance of approximately 50 cm.

### Experimental Design and Procedure

On consecutive days, participants performed two versions of the verbal and non-verbal memory tests described below. All patients performed a version of each of these tests once on and once off dopamine-replacement therapy. The ON-OFF order was counterbalanced across participants, with half of the patients performing the task first ON medication and the other half performing the first session OFF treatment. During ON testing sessions, PD patients took their dopamine-replacement medication as prescribed by their treating neurologist. During OFF testing sessions, PD patients abstained from dopamine-replacement therapy for a minimum of 12 and a maximum of 18 hours prior to testing. Control participants were also tested on these memory measures on consecutive days. Although control participants did not take dopamine-replacement medication during either testing session, their data were analyzed to parallel the ON-OFF order of the PD patient to whom they were matched based on age. Controls were matched to PD patients, and hence their ON-OFF orders were determined, prior to testing. Therefore, we controlled for order, fatigue, and possible practice effects.

#### 1. Rey Auditory Verbal Learning Test (RAVLT): Verbal Test of Explicit Memory

In each session, a version of the main List A, consisting of 15 words, was presented to participants. Words appeared one at a time in the centre of the computer screen at a rate of one word per second and participants were instructed to commit these words to memory. Immediately following the study phase of this main list, participants were asked to write down as many words as they could recall. This study-immediate recall procedure for List A was repeated three times in each session.

A version of List B, also consisting of 15 words, was then presented once with presentation parameters identical to those described for List A. Participants immediately recalled as many words as they could from List B, which served as an interference event. Next, they were asked to recall as many words as they could from the main List A. After a 30-minute delay, participants were asked to recall as many words as they could from Lists A and B without any further study presentations.

A recognition memory test followed. During this test, all of the words from Lists A and B were presented, one at a time, mixed randomly among 22 new words, in the centre of the computer screen. Participants indicated whether the word had been presented in the previous study phases or whether it was new, by pressing the ‘z’ key for ‘old’ and the ‘/’ key for ‘new’ judgements. For words judged as ‘old’, participants were additionally prompted to indicate with a key press whether the word appeared previously in List A (i.e., ‘1’), List B (i.e., ‘2’), or whether they were unsure of the source (i.e., ‘3’).

Appendix S1A provides Versions 1 and 2 of Lists A and B and the new words included in the recognition tests. For all participants, Version 1 of all lists was employed in Session 1 and Version 2 was used in Session 2, regardless of ON or OFF medication status. In this way, for half of the patients, Version 1 was tested on medication whereas for the remaining patients, Version 2 was tested on medication. Control participants' data were analyzed to correspond to the data of the PD patient to whom they were matched. In this way, we controlled for order, list, fatigue, and practice effects.

#### 2. Aggie Figures Learning Test (AFLT): Non-verbal Test of Explicit Memory

The AFLT was entirely analogous to the RAVLT save that abstract symbols were employed rather than words. In brief, these abstract symbols were presented one at a time in the centre of the computer screen for one second each. During the recall sessions, participants were asked to draw the abstract designs from memory, on blank sheets of paper. Further, five study-immediate recall trials were performed rather than three to compensate for increased difficulty in learning abstract figures relative to common words.

Appendix S1B provides examples of designs used in the AFLT.

#### 3. Measures of Encoding and Retrieval from Long-term Memory

The difference in the number of items recalled from the first to the last study-immediate recall trials in both the RAVLT and the AFLT provided a measure of learning rate or encoding [29], [30]. We predicted, as have others, that performance on study-immediate recall trials would be influenced, in addition to encoding and retrieval processes, by immediate or working memory [31], [32], all in unknown proportions. Subtracting performance on the first study-immediate recall trial from the last, permitted measurement of the proficiency and efficiency of learning, over repeated presentations, controlling for the effects of immediate memory and recall abilities [31]–[33]. The factor that is predicted to systematically increase across study-immediate recall trials is the extent to which items are transferred to more long-term memory (i.e., encoding).

In contrast, the number of items recalled on the RAVLT and AFLT from Lists A and B following a delay, is thought to preferentially index retrieval processes [34]. Recognition of studied words and abstract designs from among new items, after a delay, also was intended to stress retrieval processes [35]. Sensitivity of recognition memory for List A and B items was estimated using *d'* scores [36].

## Results

**Figure 1:**
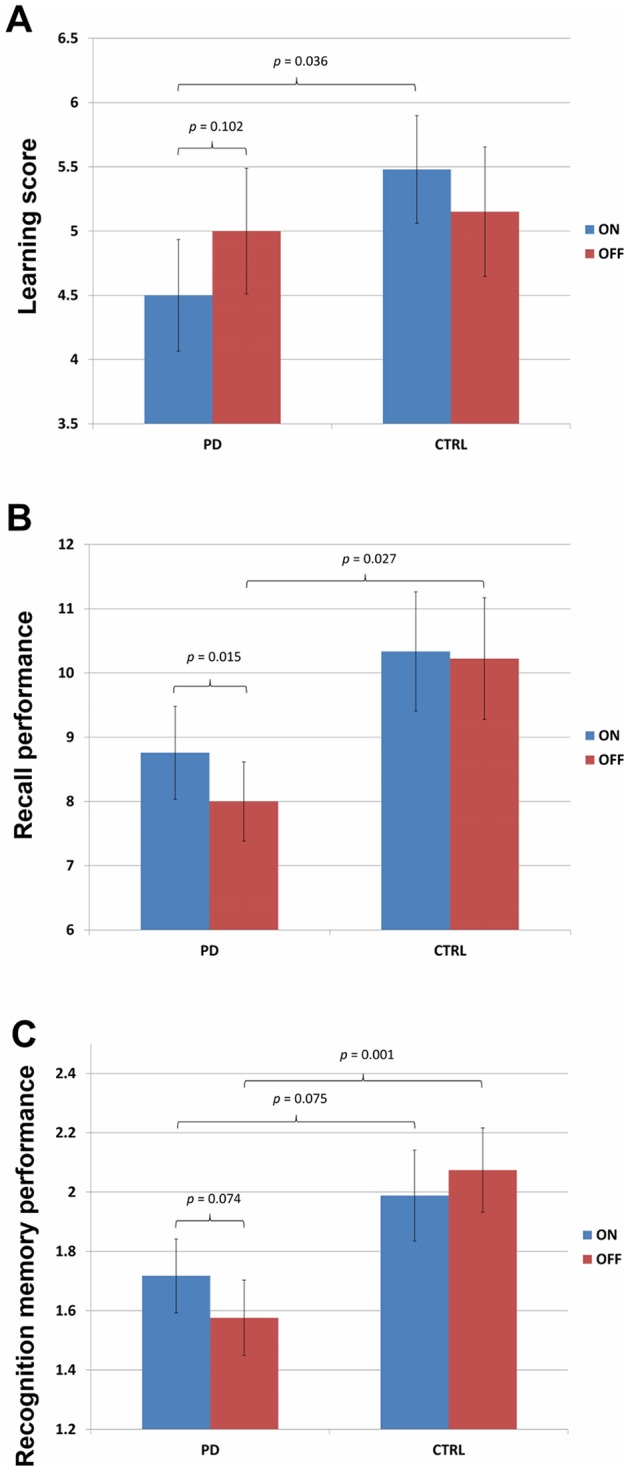
Performance Scores for PD patients and Controls in ON and OFF Sessions. Figure 1A demonstrates learning scores for PD patients and controls in ON and OFF sessions. Mean learning scores, calculated as total number of items recalled on the final minus the first study-immediate recall trial, for the RAVLT and AFTL combined, are presented for PD and controls, in both sessions separately. For PD patients, scores in the ON medication session appear in red whereas those for the OFF session are presented in blue. Although control participants did NOT receive dopaminergic therapy during any session of the experiment, their data are presented here to correspond to the ON-OFF order of the PD patient to whom they were matched. Error bars represent standard errors about the mean (SEM). Figure 1B demonstrates recall performance for PD patients and controls in ON and OFF sessions. Mean number of items recalled after delay for Lists A and B on the RAVLT and AFLT combined, are presented for PD and control participants, separated by session. For PD patients, scores in the ON session appear in red whereas those for the OFF session are presented in blue. Although control participants did NOT receive dopaminergic therapy during any session of the experiment, their data are presented here to correspond to the ON-OFF order of the PD patient to whom they were matched. Error bars represent SEM. Figure 1C demonstrates recognition memory performance for PD patients and controls in ON and OFF sessions. Mean *d'* scores for List A and B items, recognized from newly-presented items after delay, for the RAVLT and AFLT combined, are presented for PD and control participants, separately for each session. For PD patients, scores in the ON session appear in red whereas those for the OFF session are presented in blue. Although control participants did NOT receive dopaminergic therapy during any session of the experiment, their data are presented here to correspond to the ON-OFF order of the PD patient to whom they were matched. Error bars represent SEM.

Below, we report the analyses on the data for the RAVLT and AFLT, contrasting measures that we intended to index encoding versus to stress retrieval. For the statistical analyses, raw scores were converted to Z-scores to avoid differences in the scale of performance across the RAVLT and AFLT. For between-subject analyses, raw scores were normalized within session, across groups. For within-subject analyses, raw scores were normalized relative to performance of each group, across sessions. Analyses performed with raw scores produced identical patterns of results. Group averages of raw scores reflecting learning, recall, and recognition performance, and statistical outcomes of contrasts on these scores, are presented in Figure 1, Panels A to C.

### Encoding Measure

Learning scores were obtained separately for the RAVLT and AFLT, as well as for each group (PD vs. Control), in each session (ON vs. OFF). Learning scores were calculated as number of correctly recalled items for main Lists A on the final study-immediate recall trial (i.e., Trial 3 for the RAVLT and Trial 5 for the AFLT) minus number of correctly recalled items on study-immediate recall Trial 1. These scores are presented for both groups, in each session in Table 2. Figure 1A presents the learning scores for PD patients and controls, on the RAVLT and AFLT combined, in the on and off sessions.

**Table 2:** Learning in the RAVLT and AFLT for PD and control participants in both experimental sessions.

	PD			Control		
	First trial	Final	Learning scores	First trial	Final trial	Learning scores
RAVLT ON	5.48 (0.32)	9.00 (0.47)	3.52 (0.43)	6.33 (0.50)	10.70 (0.45)	4.37 (0.37)
RAVLT OFF	5.14 (0.34)	9.03 (0.44)	3.90 (0.35)	6.66 (0.54)	10.59 (0.44)	3.93 (0.47)
AFLT ON	2.31 (0.31)	7.80 (0.58)	5.48 (0.44)	2.79 (0.31)	9.33 (0.66)	6.59 (0.47)
AFLT OFF	1.90 (0.25)	8.01 (0.63)	6.10 (0.54)	3.04 (0.35)	9.41 (0.64)	6.37 (0.54)

Mean number of items recalled (SEM) on the first and final immediate-recall trials in the RAVLT and AFLT are presented separately for PD and control participants in the ON and OFF medication Sessions. Learning scores, calculated as total number of items recalled on the final minus the first study-immediate recall trial, also appear. Scores for control participants are also displayed according to the ON-OFF order of the PD patients to whom they were matched even though no healthy controls were treated with dopaminergic medication at any time.

### Retrieval Measures

The number of words and abstract designs recalled in the RAVLT and the AFLT respectively from the main Lists A and the interference Lists B, following a 30-minute delay, for PD patients and controls, in both sessions, appear in Table 3. Figure 1B presents the average number of items recalled after delay on the RAVLT and AFLT combined, in the on and off sessions, for both PD patients and controls.

**Table 3:** Measures of long-term memory on the RAVLT and AFLT, for PD patients and controls, in both experimental sessions.

		PD		Control	
		Recall after delay	*d'*	Recall after delay	*d'*
**RAVLT**	**List A ON**	6.241 (0.513)	2.171 (0.118)	7.815 (0.602)	2.473 (0.172)
	**List A OFF**	5.931 (0.446)	2.09 (0.136)	7.889 (0.545)	2.592 (0.149)
	**List B ON**	1.31 (0.217)	1.124 (0.113)	2.148 (0.482)	1.453 (0.172)
	**List B OFF**	1.31 (0.244)	0.89 (0.103)	1.778 (0.46)	1.487 (0.155)
**AFLT**	**List A ON**	8.379 (0.609)	2.327 (0.118)	9.074 (0.654)	2.381 (0.124)
	**List A OFF**	7.862 (0.65)	2.256 (0.118)	9.222 (0.731)	2.606 (0.121)
	**List B ON**	1.586 (0.327)	1.249 (0.149)	1.63 (0.321)	1.646 (0.145)
	**List B OFF**	0.897 (0.224)	1.069 (0.152)	1.556 (0.33)	1.613 (0.144)

The mean number of items recalled out of a total of 15 for Lists A and B after delay, along with the mean *d'* scores, reflecting sensitivity of old-new discriminations for Lists A and B on both the RAVLT and AFLT are presented. Scores for PD patients appear separately for ON and OFF medication sessions. Control participants' data are displayed to correspond to the ON-OFF order of the PD patient to whom they were matched, although they were not treated with dopaminergic medication at any time.

Sensitivity in distinguishing studied words and abstract designs from new items, estimated by *d'* scores [36] for List A and B items, on the RAVLT and the AFLT, for PD patients and controls in both sessions, are presented in Table 3. Figure 1C compares mean *d'* scores across sessions on the RAVLT and AFLT combined, for PD patients and controls.

### Encoding and Retrieval Between Groups

#### 1. Encoding Measure

We performed 2×2 ANOVAs on normalized learning scores, with Group (PD vs. Control) as the between-subject factor and Test type (Verbal/RAVLT vs. Non-verbal/AFLT) as the within-subject variable, for ON and OFF sessions separately. Although, control participants did not take dopaminergic medication at any point in this study, their data were analyzed to parallel the ON and OFF session of the PD patient to whom they were matched. We found significantly poorer learning for PD patients relative to controls in the ON session, *F* (1, 54)  = 4.60, *MSe*  = 1.09, *p*<0.05 but no significant group difference in the OFF session, *F*<1. All other main effects and interactions were not significant, all *F*<1.

#### 2. Retrieval Measures: Recall

We performed 2×2 ANOVAs on normalized recall scores, with Group (PD vs. Control) as the between-subject factor and Test type (Verbal/RAVLT vs. Non-verbal/AFLT) as the within-subject variable, for ON and OFF sessions separately. The main effect of Group was significant in the OFF session only, *F* (1, 54)  = 6.23, *MSe*  = 1.30, *p*<0.025, reflecting poorer recall for PD patients compared to controls. The effect of Group was not significant in the ON session, *F* (1, 54)  = 2.72, *MSe*  = 1.33, *p* = 0.105. In both sessions, the main effect of Test type, *F*<1, and the Group x Test type interactions, *F* (1, 54)  = 1.16, *MSe*  = 0.58, *p*>0.250 and *F* (1, 54)  = 1.36, *MSe*  = 1.36, *p*>0.225, for ON and OFF respectively, were also not significant.

#### 3. Retrieval Measures: Recognition

Analogous 2×2 ANOVAs, with Group (PD vs. Control) as the between-subject factor and Test type (Verbal/RAVLT vs. Non-verbal/AFLT) as the within-subject variable, for ON and OFF sessions separately, were performed on normalized recognition memory scores. The main effect of Group was significant in the OFF session only, *F* (1, 54)  = 6.23, *MSe*  = 12.71, *p*<0.001, reflecting poorer recognition memory for PD patients compared to controls. The main effect of Group in the ON session was marginally significant, *F* (1, 54)  = 3.31, *MSe*  = 1.24, *p* = 0.075, suggesting a trend toward poorer recognition memory performance for PD patients compared to controls. The effect of Test type and the Group × Test type interactions in both sessions were not significant, all *F*<1.

#### 4. Summary

Off medication, learning scores were comparable for PD patients and control participants. On medication, PD patients' learning was impaired relative to that of controls. In contrast, off medication, PD patients' recall and recognition memory was significantly poorer than that of controls. On medication, PD patients' and control participants' recall was equivalent, and there was only a trend toward poorer recognition in PD patients compared to controls.

### Encoding and Retrieval within Groups

#### 1. PD Group

On PD patients' normalized learning, recall, and recognition scores, we performed a 3×2×2 ANOVA, with Experimental phase (Learning vs. Delayed Recall vs. Recognition), Test type (Verbal/RAVLT vs. Non-verbal/AFLT), and Session (ON vs. OFF medication) as within-subject variables. Only the Experimental phase × Session interaction was significant, *F* (2, 56)  = 3.99, *MSe*  = 0.47, *p*<0.025. All other interactions and main effects were not significant, all *F*<1.

To better understand the Experimental phase × Session interaction, we examined the effect of Session on the measures expected to index encoding and those intended to stress retrieval, separately. First, we performed a 2×2 ANOVA on the normalized learning scores with Test type (Verbal/RAVLT vs. Non-verbal/AFLT) and Session (ON vs. OFF medication) as within-subject variables. The main effect of Session did not reach significance, *F* (1, 28)  = 2.49, *MSe*  = 0.51, *p* = 0.126. The main effect of Test type and the Test type × Session interaction were also not significant, both *F*<1.

Next, we performed a 2×2×2 ANOVA on the normalized recall and recognition scores with Experimental phase (Recall vs. Recognition), Test type (Verbal/RAVLT vs. Non-verbal/AFLT), and Session (ON vs. OFF medication) as within-subject variables. Only the main effect of Session was significant, *F* (1, 28)  = 8.46, *MSe*  = 0.30, *p*<0.005, reflecting poorer recall and recognition memory scores off relative to on medication. The main effects of Experimental Phase, Test type, and all interactions were not significant, all *F*<1.

#### 2. Control Group

Analogous ANOVAS were performed on control participants' normalized learning, recall, and recognition performance scores. Again, control participants did not take dopaminergic medication at any point in this study. However, their data were analyzed to parallel the ON and OFF session of the PD patient to whom they were matched. The 3×2×2 ANOVA, with Experimental phase (Learning vs. Delayed Recall vs. Recognition), Test type (Verbal/RAVLT vs. Non-verbal/AFLT), and Session (ON vs. OFF medication) as within-subject variables, revealed no significant main or interaction effects, all *F*<1.

To mirror the analyses performed on PD patients' data, we also examined the effect of Session on the measures expected to index encoding and to stress retrieval separately for control participants. The 2×2 ANOVA on the normalized learning scores with Test type (Verbal/RAVLT vs. Non-verbal/AFLT) and Session (ON vs. OFF medication) as within-subject variables and the 2×2×2 ANOVA on the normalized recall and recognition scores with Experimental phase (Recall vs. Recognition), Test type (Verbal/RAVLT vs. Non-verbal/AFLT), and Session (ON vs. OFF medication) as within-subject variables, both uncovered no significant main or interaction effects, all *F*<1.

#### 3. Summary

For PD patients, differences in learning performance related to dopaminergic medication status, did not reach significance. However, dopaminergic medication significantly improved retrieval as assessed by delayed recall and recognition memory performance. There were no significant differences between ON-OFF sessions for control participants.

## Discussion

Off medication, PD patients performed comparably to controls on a measure that indexed encoding (i.e., standardized learning difference score) but more poorly on indices that preferentially reflected retrieval processes (i.e., standardized recall and recognition after delay). On dopamine replacement medication, learning rate was poorer for PD patients compared to controls. In contrast, delayed recall and recognition memory performance was improved by dopamine replacement medication. This pattern of findings arose with both verbal and non-verbal test materials. This constitutes the first study to show dissimilar effects of dopaminergic medication on different aspects of memory.

Most previous investigations of memory have tested PD patients on medication only. Our findings caution that these results represent the summed effects, in unknown proportions, of some deficient and other spared baseline memory processes, as well as medication-induced improvements in some operations and impairments in others. The resulting confounded estimate of memory performance is expected to vary across experiments with even small methodological changes, if the contributions of encoding and retrieval are differentially emphasized, potentially accounting for the inconsistency of the PD memory literature [9], [12], [37]. Two previous studies investigated recognition memory both on and off medication in PD but did not attempt to dissociate encoding and retrieval processes [10], [24]. Bronnick and colleagues [11] did test learning and retrieval independently but they only examined medication-naive PD patients. At odds with our results, they found impaired rate of learning for PD patients relative to controls off medication. The reason for this discrepancy is not entirely clear, given that we employed similar methodology. Because we tested performance both off and on medication, however, and observed dissociated effects of PD and medication on learning rate versus later recall and recognition, we directly refute their conclusion that in PD, previously-demonstrated memory impairments are attributable to encoding deficiencies. Specifically, we found that retrieval deficits were maximal in the session where learning rate was equivalent for PD patients and controls (i.e., in the OFF session). Conversely, retrieval performance was improved in the ON session, in which learning rate was impaired for PD patients relative to controls. Our study constitutes the first systematic investigation of encoding and retrieval in PD, and of the differential effect of dopamine replacement on these separate processes.

### Interpretation of Findings Related to Encoding

Functions that are normal off medication and worsened by dopaminergic therapy in PD have been shown previously to depend upon VTA-innervated brain regions [5], [38], [39]. The VTA is relatively spared in PD, especially early in disease, and its efferent brain regions are adequately supplied with dopamine off medication [1]. It is hypothesized that these brain regions are overdosed by dopamine-replacement levels that are therapeutic for dorsal striatum-mediated motor symptoms [40]. Combining neuroimaging and behavioural tests in PD patients on and off medication, this overdose theory has been supported empirically. Dopaminergic medication-related decreases have been observed in ventral striatum [5], [38], in ventromedial prefrontal cortex and posterior insula [39], as well as in orbitofrontal cortex [38]. VTA-innervated hippocampus, that is known to be critical for explicit memory encoding, might also be overdosed by dopaminergic therapy in PD, potentially accounting for our findings. This would be in keeping with observed regulation of hippocampal-ventral striatum-globus pallidus-mediated learning by VTA in healthy participants [41], [42].

That PD patients learned lists of words and abstract images *more poorly* than controls *on* dopaminergic medication, but equivalently at baseline, seems to contradict a larger literature suggesting that dopamine improves learning in non-human animals [43], [44] and in healthy human participants [45], [46]. The current experiment differs from many of these experiments in a few important respects. These differences and how they might account for apparent discrepancies with respect to dopamine's effects on learning and memory will be discussed in the paragraphs that follow.

First, typical learning paradigms confound learning and performance. Proficiency of learning motor acts or skills, or of encoding associations between stimuli and rewards, and between stimuli and responses, is usually confirmed by asking participants to perform the trained actions or enact decisions referring to the learned associations. If dopamine improves skill performance or decision making, this can simulate improved learning. Atallah and colleagues elegantly demonstrated this fact in testing the hypothesis that the dorsal striatum mediates stimulus-response and response-reward association learning [47]. They sought to distinguish *learning* these relations from *performing* appropriate response selections based on what was learned. In awake and ambulatory rats, first they compared the effect on performance in the training session of infusing the dorsal striatum with a) a gamma-aminobutyric acid (GABA) agonist in one group – expected to have inhibitory effects, and b) saline in another – expected to have no effect on dorsal striatum function. They found that GABA infusions to dorsal striatum impaired rats' ability to consistently select a rewarded relative to an unrewarded arm in a y-maze task on the basis of odour cues during the training session. This seemed to suggest that inhibiting dorsal striatum function impaired learning relations. However, when they stopped the GABA and saline infusions during a later test phase, animals in the experimental group performed equivalently to control animals. This revealed that stimulus-response and stimulus-reward associations had been learned equally well during the training session for both groups, but suppressing dorsal striatum function had interfered with enactment of correct responses. Conversely, and to drive home the point, in another experiment, when GABA agonist versus saline infusions to the dorsal striatum were instituted at test only, performance became impaired for the GABA group relative to the saline group despite identical learning for both groups during training.

A closer examination of a representative investigation of the effect of dopamine on learning in healthy human participants seems to echo the results of Atallah and colleagues [47]. Knecht and colleagues [45] investigated the effect of one Levodopa/Carbidopa 100/25 mg tablet per day on implicit learning in healthy adults. Participants learned to implicitly associate auditorily-presented pseudowords with object drawings, through trial and error with feedback. They performed 400 learning-feedback trials per day over five days. On Day 1 (i.e., over the course of 400 trials), there was no difference in the percentage of correct pseudoword-drawing pairs learned by participants receiving Levodopa versus placebo. In fact, significant differences between groups were not evident until the end of Day 2 (i.e., at 800 trials), when learning exceeded 70% correct. If dopamine's role is to facilitate learning, maximal effects would be expected during the steepest part of the learning curve (i.e., over Days 1 and 2). In this study, however, the effect of dopamine was greatest when learning had reached a plateau and performance accuracy ranged between 80–90%. This pattern of findings seems more consistent with Levodopa-mediated improvement in *retrieval* of pseudoword-drawing associations than on learning these associations *per se*. This revised interpretation of these results is entirely consistent with our findings in PD patients that dopamine-replacement medication improves retrieval.

In the current experiment, we aimed to distinguish encoding of verbal and non-verbal materials from other influences on memory performance. The RAVLT and AFLT methodology, with repeated study-immediate recall events that use the same stimuli across trials, permits a less confounded estimate of encoding processes. Performance on each study-immediate recall trial reflects the combined influences of word or figure encoding and retrieval from long-term memory, as well as immediate or working memory processes. However, the number of items transferred to long-term memory is expected to systematically increase across study-immediate recall trials with less clearly predictable effects on other processes. Consequently, subtracting performance in the final from the first stimulus-recall trial provides a less confounded estimate of encoding or learning [31]–[33], [48]–[51]. Attempting to isolate encoding from other influences on memory is particularly important given that immediate or working memory processes have been shown to improve with dopaminergic medication in PD [52]–[60] and in healthy young adults [61]–[66].

Dosages of dopaminergic medications in our study and those investigating dopamine's effect on learning in healthy adults differ greatly. This is another potentially important difference. In the current study, the average daily Levodopa equivalent was 520 mg compared to 100 mg in the study performed by Knecht and colleagues [45]. PD patients are also arguably more susceptible to overdose effects from exogenous dopaminergic medications because dopamine-producing neurons also regulate synaptic dopamine [67]. As these cells are lost in PD, so is dopamine buffering capacity.

Although our result of poorer learning for PD patients on dopaminergic medication relative to healthy control participants might on the surface seem at odds with findings in non-human animals and healthy human participants, it is entirely in keeping with findings in PD. Our results, however, extend the learning situations that are impaired by dopaminergic medication to include intentional and explicit encoding of word and image lists. That is, in PD, learning is the function most frequently worsened by dopamine replacement therapy [68]–[73]. A number of studies have revealed deficits related to dopamine replacement medications in probabilistic associative learning in PD patients who perform equivalently to controls off medication [69], [72], [74]. Shohamy and colleagues [71] found that dopaminergic medication impaired learning of an incrementally-acquired, concurrent discrimination task, whereas off medication, PD patients performed as well as controls. Sequence learning was reduced for PD patients on medication [68], [70], [75], [76]. Dopamine supplementation yielded reduced facilitation for consecutive, consistent stimulus-stimulus pairings in a selection task compared to normal facilitation when PD patients were tested off medication [4]. Once stimulus-reward associations have been learned, reversing these associations is also performed more poorly for PD patients on dopamine replacement therapy [6], [77]–[83]. Dopamine therapy has been shown to impair learning from negative feedback [84].

Finally, there are a number of studies with healthy adults in which dopaminergic therapy has led to diminished learning. Breitenstein and colleagues [85] found that administering a dopamine agonist significantly impaired novel word learning in healthy volunteers compared to placebo. Similarly, Pizzagalli and colleagues [86] and Santesso and colleagues [87] found that reward learning was impaired in healthy adults after pramipexole was administered.

### Interpretation of Findings Related to Retrieval

The SN, which is significantly degenerated at even the earliest stages of clinical PD, has dopamine projections nearly exclusively to the dorsal striatum. Hence, the pattern of baseline deficits in early PD that are remediated by dopamine supplementation in PD is the signature of a dorsal striatum-mediated process. In our study, PD patients were impaired at baseline but improved with dopamine replacement on all measures that stressed retrieval, even though encoding was impaired in PD patients relative to control in the on session. Our results are consistent with a role for dorsal striatum in retrieval. A review of the literature reveals evidence in line with these findings.

Surveying cognitive deficits in patients with dorsal striatum lesions in fact reveals that the most common impairment is in *explicit* memory [88]–[90]. In functional neuroimaging studies, dorsal striatum is preferentially activated for learned relative to random motor sequences [91], for familiar items in an episodic recognition test [20], and while recalling recently-learned category membership [92], [93]. Unlike activity in ventral striatum that tracks the progression of learning, dropping off as performance asymptotes and hence learning plateaus [4], [91], [93], preferential activation of the dorsal striatum in neuroimaging studies persists well after sequences or categorization rules [93] have been encoded. Together these findings suggest that although dorsal striatum is implicated in explicit memory function, it does not underlie learning or encoding processes. Based on our data, we posit that it mediates retrieval of previously encoded information.

## Conclusion

Increasingly, cognitive impairment is identified as a significant cause of disability in PD [94]. The nature and pathophysiology of cognitive deficits in PD are not fully understood. Enhanced understanding of these impairments and the effect of dopamine replacement therapy on cognition in PD is an important aim as it will translate directly into improved clinical care. Here we show that learning rate and retrieval processes are oppositely affected by PD and dopaminergic medication. Whereas encoding rate is spared at baseline in PD, successful retrieval from long-term memory is impaired. On dopaminergic medications, PD patients encoded both verbal and non-verbal information more poorly than controls whereas dopamine replacement improved later retrieval from long-term memory. These findings shed light on the memory literature in PD and subsequent studies will aim to better understand the brain regions that mediate these findings. More specific understanding of memory impairments in PD might guide clinicians to consider a broader range of symptoms and tailor medication strategies to specific patient complaints and priorities.

## Supporting Information

Appendix S1 **Word list and images used in tests of explicit memory.** Appendix S1A contains all words used in the Rey Auditory Verbal Learning Test (RAVLT) for each experimental phase for Sessions 1 and 2. Appendix S1B contains example images used in the Aggie Figures Learning Test (AFLT).
(DOC)
