# Supplementary material for: Differential Effects of Parkinson's Disease and Dopamine Replacement on Memory Encoding and Retrieval
Source: PLoS One. 2013 Sep 26;8(9):e74044. doi: 10.1371/journal.pone.0074044 (PMC3784427; doi:10.1371/journal.pone.0074044)
Supplement: Appendix S1 — Word list and images used in tests of explicit memory. Appendix S1A contains all words used in the Rey Auditory Verbal Learning Test (RAVLT) for each experimental phase for Sessions 1 and 2. Appendix S1B contains example images used in the Aggie Figures Learning Test (AFLT). (DOC) [file pone.0074044.s001.doc]

| **Appendix 1A**  RAVLT Stimulus Sets | | | |
| --- | --- | --- | --- |
| **Session 1** | | **Session 2** | |
| **Recall** | **Interference** | **Recall** | **Interference** |
|  |  |  |  |
| Doll | Dish | Violin | Orange |
| Mirror | Jester | Tree | Table |
| Nail | Hill | Scarf | Toad |
| Sailor | Coat | Ham | Corn |
| Heart | Tool | Suitcase | Bus |
| Desert | Forest | Cousin | Chin |
| Face | Perfume | Earth | Beach |
| Letter | Ladder | Stairs | Soap |
| Bed | Girl | Dog | Hotel |
| Machine | Foot | Banana | Donkey |
| Milk | Shield | Town | Spider |
| Helmet | Pie | Radio | Money |
| Music | Insect | Hunter | Book |
| Horse | Ball | Bucket | Soldier |
| Road | Car | Field | Padlock |

**Appendix 1B**

Example AFLT Figures
